# Analysis of the Sensory Profile and Physical and Physicochemical Characteristics of Amazonian Cocoa (*Theobroma cacao* L.) Beans Produced in Different Regions

**DOI:** 10.3390/foods13142171

**Published:** 2024-07-09

**Authors:** Renato Meireles dos Santos, Niara Maria de Jesus Silva, Fábio Gomes Moura, Lúcia de Fátima Henriques Lourenço, Jesus Nazareno Silva de Souza, Consuelo Lúcia Sousa de Lima

**Affiliations:** 1 Center for Valorization of Amazonian Bioactive Compounds (CVACBA), Federal University of Pará, Avenida Perimetral da Ciência, km 01-Guamá, Belém 66075-750, Pará, Brazil; remeisan3@gmail.com (R.M.d.S.); n.alimentoss@gmail.com (N.M.d.J.S.); fabiogmoura5@gmail.com (F.G.M.); jsouza@ufpa.br (J.N.S.d.S.); 2 Graduate Program in Food Science and Technology (PPGCTA), Federal University of Pará, Rua Augusto Corrêa S/N, Guamá, Belém 66075-900, Pará, Brazil; sousa@ufpa.br

**Keywords:** Eastern Amazon, sensory analysis, Amazonian cocoa, Quantitative Descriptive Analysis, sensory profile

## Abstract

The cocoa tree (*Theobroma cacao* L.) has seeds that after processing become a valuable agricultural commodity in the world. In Brazil, the state of Pará located in the Eastern Amazon is the main producer, accounting for more than 53% of the national production. Although the region is the largest producer, no studies are found in the literature containing data on the sensory quality of the beans. Thus, the purpose of this study is to establish the sensory profile of Amazonian cocoa from the main cocoa-producing regions of the Eastern Amazon (Lower Tocantins River, Northeast, West, Southeast and Trans-Amazon). The sensory profile was established from the Quantitative Descriptive Analysis (QDA), in addition to using an affective test to verify consumer preference for the chocolates produced. Physical, physicochemical and bioactive compound evaluations were carried out. Beans from different regions had a similar sensory profile; however, it was possible to observe some differences in certain descriptor terms. In the physical evaluation, the samples presented an acceptable commercial standard, and the humidity was within the values established by the legislation. It was found that the post-harvest and/or processing parameters had no influence on the quality attributes of cocoa beans.

## 1. Introduction

The cocoa tree (*Theobroma cacao* L.) has seeds that after being processed become a valuable agriculture commodity, as it is the main raw material used for the production of chocolates and derivatives. More than 58 countries are involved in cocoa production, many of which rely heavily on exports for their economic development [1].

The worldwide demand for beans has generated higher demands regarding their quality, which involves complex criteria based on the national [2] and international laws, ISO 2451 [3], and the requirements of expert buyers who buy beans from fine origins directly for specific chocolate companies, which take into account extrinsic and intrinsic attributes [4]. However, for production of fine chocolates, the sensorial particularities are important and determined by the flavor, which are related to a set of genotypic and edaphoclimatic factors and pre-processing and processing practices. The relationship of all these factors guarantees the final product a pattern of its own identity [5].

In Brazil, the state of Pará located in the Eastern Amazon is the main producer, accounting for more than 53% of the national production in 2020 [6]. Taking into account the territorial extension, there are five cocoa-producing regions (Lower Tocantins River, Northeast, West, Southeast and Trans-Amazon). Cocoa beans can have several characteristics according to geographical conditions, a characteristic terroir.

Obtaining sensory parameters of these beans is important not only for characterization but is also essential to ensure the quality of this product on the market. In addition, the regional characterization of these beans must be carried out taking into account the great botanical diversity and variation in each region [7].

In this sense, sensory analysis is an efficient tool used in the evaluation of products in the food area. It is a scientific methodology indicated to measure, evaluate and interpret human reactions related to the characteristics of products as they are perceived through the senses (touch, smell, taste, vision and hearing), minimizing potential effects such as the brand and other information that may influence the consumer perception. Sensory techniques can be applied by trained evaluators or consumers, with reference materials linked to the characteristics of the product under study [8,9].

Among the sensory tests, the Quantitative Descriptive Analysis (QDA) stands out, which is considered one of the most robust techniques for providing a complete description of all the sensory properties of a product, taking into account all the perceived sensations (visual, auditory, olfactory, gustatory and tactile), resulting from an extensive statistical analysis of the data. In addition, it allows for the development of the sensory profile, based on the perception of trained evaluators, who evaluate the products qualitatively and quantitatively and aim to quantify the intensity of the perceived attributes [8,10].

As cocoa-derived products are consumed all over the world and have a high market value, their sensory characteristics are continuously investigated, and many studies show that the attributes of aromas and flavors such as acidity, bitterness, woody astringent, green, fruity, floral, nut, cocoa, chocolate, smoked, burnt, moldy and animal are the most evaluated by sensory panels [11,12,13,14].

Thus, there is a need to carry out a sensory evaluation of this Amazonian raw material, in order to be able to differentiate it according to its quality, adding value and identity, granting it a market differential based on the characteristics of its origin. 

## 2. Material and Methods

### 2.1. Raw Material

A total of 35 commercial samples of beans (ranging from 0.5 kg to 3 kg) of Amazonian cacao fermented and dried by producers from the five main producing regions in the Eastern Amazon (Lower Tocantins River 6, Southeast 7, West 3, Northeast 4 and Trans-Amazon 15) were collected. After collection, the samples were tagged and stored at −4 °C for further analyses.

### 2.2. Physical and Physicochemical Description of Beans 

For the cutting test [3], beans (100 units/of each sample) were cut lengthwise with pruning shears. Half of each bean was placed on a classification board and, by visual inspection, the counts were performed according to color (brown, partially brown, violet, white and slate), with the degree of compartmentalization of the cotyledons (presence or absence) and in relation to the presence of defects (moldy, smelling of smoke, damaged by insects, slate, sprouted and flattened) [15].

After inspection, with the aid of a stainless steel cutting tool, the beans were manually peeled to separate the nibs (cotyledons), shell and germ. Subsequently, the nibs were crushed in a crusher (model LUCA-226/5) and kept in a freezer at −18 °C for future analysis.

#### 2.2.1. Moisture

The moisture content of the fermented and crushed nibs was determined by gravimetric method number 925.09 [16], in an oven at 105 °C with air circulation (model Q314M252, Quimis, São Paulo, Brazil).

#### 2.2.2. Titratable Acidity

Titratable acidity was determined, according to IAL [17], by potentiometric titration with a digital pH meter (model LUCA-210, Piracicaba, Brazil) using 0.1 N sodium hydroxide solution. 

#### 2.2.3. Water Activity (aW)

Water activity was quantified through water fugacity, using the dielectric constant in AQUALAB equipment (Decagon Devices, Inc.—Pullman, WA, USA) in triplicate.

### 2.3. Bean Roasting

The beans were roasted at 120 °C for 60 min in an oven with air circulation (model Q314M252, Quimis, São Paulo, Brazil), then the nibs and the shell were broken and separated. The nibs were crushed (Tramontina grinder, 1800 W motor) until a powder was obtained and submitted to sensory analysis.

### 2.4. Quantitative Descriptive Analysis

The Quantitative Descriptive Analysis (QDA) was performed according to the parameters recommended by Brazilian Association of Technical Standards (ABNT)—NBR ISO 13299 [18], following the methodology described by Stone and Sidel [19]. 

For the selection of sensory panelists, different sensory tests were carried out (recognition of odors, basic flavors and threshold). Then, discriminative tests (triangular and duo-trio) and descriptive tests with the wheel of aromas and flavors [20] were used. A total of 11 sensory panelists were selected (8 women and 3 men, aged between 18 and 45 years) who carried out a survey of nine aroma attributes (cocoa, acidity, sweet, fruity, floral, spice, wood, smoke and animal) and ten attributes of flavor (cocoa, acidity, sweet, fruity, floral, spice, wood, astringent, bitter and smoke), using reference materials of selected attributes for familiarization. After training, the panel was able to identify and quantify the sensory attributes of cocoa and chocolate, with performance being evaluated in terms of repeatability, reproducibility and consensus.

For the final evaluation of the samples, unstructured 9 cm scales were used. The evaluation of the powder nibs as well as the training of the panel were carried out at the Sensory Analysis Laboratory of the Center for the Valorization of Bioactive Compounds in the Amazon at the Federal University of Pará (CVACBA/UFPA). The research project was approved by the Research Ethics Committee of the Federal University of Pará (UFPA), Reference number 4031506.

### 2.5. Chocolate Production

For each region, the nib samples were crushed and mixed, producing a chocolate with 70% cocoa (nibs) and 30% refined sugar (Cia. União). Chocolate production consisted of the refining stage in a stone mill (Premier, SL.NO: C1R0302N18, 200W) with the addition of nibs and sugar, which remained for 24 h until obtaining the liquor. Subsequently, the liquor was subjected to tempering (Temperadeira, Universal brand) to control crystallization in order to stabilize it; then, the chocolates were molded and refrigerated at 10 °C until the following day, when the acceptance test was carried out. 

### 2.6. Acceptance Test

The chocolates produced (1 per region) were submitted to an acceptance test, with 99 tasters (53 men and 46 women) who are consumers of products derived from cocoa, who evaluated the following attributes: aroma, flavor, bitterness, acidity, texture, astringency and overall impression. For the test, a structured 9-point hedonic scale was used, anchored by the following terms: 9 “I liked it very much”, 8 “I liked it a lot”, 7 “I liked it moderately”, 6 “I liked it slightly”, 5 “I neither liked nor disliked it”, 4 “I disliked it slightly”, 3 “I disliked it moderately”, 2 “I disliked it much” and 1 “I disliked it very much” [12]. 

### 2.7. Chemical Analysis of Nibs

#### 2.7.1. Obtaining Raw Extract

The extraction took place according to the methodology proposed by Counet and Collin [21], with modifications, where 1 g of nibs was used in 19 mL of the extracting solution containing acetone, water and acetic acid (70:29.5:0.5 *v*/*v*). The samples, in solution, were shaken in Vortex (Basic Kasvi—Brazil) for 1 min. Then, it was transferred to Eppendorf tubes followed by centrifugation (CT15RE—Japan) at 8000 RPM for 20 min at 4 °C. The supernatant extract was stored at −18 °C until analysis.

#### 2.7.2. Determination of Total Polyphenols

The determination was performed by the Folin–Ciocalteu method [22] which is based on the common reactivity of polyphenols when placed in contact with phosphomolybdic and phosphotungstic acids for 30 min. The reading of the samples was performed at 735 nm in a spectrophotometer IL-592 (Kasuaki—Japan). The analysis was performed in triplicate and the results expressed in milligrams of catechin equivalents per gram of dry nibs (mg CE/g).

#### 2.7.3. Determination of Proanthocyanidins

The determination of proanthocyanidins was performed by the butanol-HCl (hydrochloric acid) method proposed by Julkunen-Tiitto [23]. This method is based on the hydrolysis of proanthocyanidins by butanol in an acid medium (HCl) under heating (90 °C), which are converted into the corresponding anthocyanins. Results were expressed in milligrams of cyanidin equivalents per gram of dry nibs (mg CyE/g).

#### 2.7.4. Determination of Astringency 

The determination of astringency was performed using the method proposed by Horne, Hayes and Lawless [24], which is based on measuring the turbidity caused by the formation of complexes between proanthocyanidins and proteins rich in proline, present in human saliva. Turbidity was determined using a TB1000 turbidimeter (Tecnopon—Brazil). The equipment calibration was performed with formazine standards (Tecnopon—Brazil) in different concentrations. Results were expressed in Nephelometric Turbidity Units (NTUs) per gram of dry nibs (NTU/g).

### 2.8. Statistical Analysis 

The results obtained in the physical, physicochemical and chemical analyses were submitted to Analysis of Variance (ANOVA) and the Tukey test to compare means in STATISTICA 7.0 (StatSoft, 2004). The results obtained from QDA were subjected to principal component analysis (PCA) using The Unscrambler^®^ X v10.4 software (CAMO Software AS 2016) where the sample attributes were used as dependent variables and the regions as independent variables. To compare means and generate graphs of the affective test results, Microsoft Excel was used.

## 3. Results and Discussion

### 3.1. Physical and Physicochemical Characterization of Cocoa Beans

The results obtained in the cutting test and in the physicochemical analyses are shown in Table 1. The cut test demonstrates that the analyzed batches were well fermented with compartmentalization and typical brown coloration [25] and corroborate with the Federation of Cocoa Commerce (FCC) [26], which considers that a batch should contain less than 5% slate/violet beans. All evaluated regions showed compartmentalization and brown coloration above 86% and 95%, respectively, while less than 1.5% were violet/slate. The values for white beans were also less than 1%, which demonstrates the uniformity of the foreign variety cultivated in the region.

It was also verified, after the longitudinal cut of the beans, that the absence of strange odors and the sum of the defects analyzed individually (moldy, smoke, damaged by insects, slate, germinated and flattened) presented values varying between 0.6 and 1.76%. Therefore, despite the values provided for in the legislation [2] addressing the percentage of defects individually, in this research, the highest sum of defects found was in the Lower Tocantins River region (1.76%), but they were still classified as type 1 beans.

The maximum humidity value found was 6.11% for the Lower Tocantins River region; thus, all are within the values recommended by legislation [2], which specifies a maximum percentage of 8%. Beans with a low moisture content avoid enzymatic reactions that affect the quality and growth of fungi that can produce undesirable metabolites during storage, such as mycotoxins [27].

It is known that the acidity of the beans initially depends on the presence of citric acid, but after fermentation and due to the action of the microorganisms that intervene in it, the acetic and lactic acids produced are what make up the majority of the acidity of the fermented and dried beans [28]. The acidity results obtained ranged from 0.21 to 0.48 Aa/100 g, where the Lower Tocantins River differed by 5% from the West and the Trans-Amazon regions. Djikeng et al. [29] evaluated cocoa beans from Cameroon and obtained low acidity values, as found for beans from the state of Pará in this research.

Water activity values (aw) directly influence the multiplication of bacteria and mainly fungi that can develop in low percentages and produce mycotoxins. The multiplication of *Aspergillus ochraceus* and *Aspergillus parasiticus* has already been verified at a minimum aw of 0.78, and for the production of mycotoxins by Aspergillus, it is necessary to have an aw greater than 0.85 [30,31]. The results obtained in this research indicate insufficient aw availability for the development of fungi; consequently, there is no risk of mycotoxin production.

### 3.2. Sensory Profile 

Sensory characteristics are important attributes for a consumer to purchase a food product. For fine chocolate, these attributes are even more demanding; therefore, the sensory characteristics related to the aroma and flavor presented by cocoa beans vary according to several parameters, such as terroir, edaphoclimatic factors, varieties, etc., and processing techniques such as fermentation, drying and roasting are important for aroma and flavor development [32,33,34]. Thus, attributes such as cocoa, acidity, bitterness and astringency must be evaluated, as well as the search for particular flavors such as floral, woody, fruity, etc. Undesirable aromas and flavors (off flavors), such as smoke, musty, dirty and animal, should also be analyzed, as these attributes devalue cocoa products [35].

Table 2 presents the values of the sensory attributes of the Amazonian cocoa bean aromas by region, obtained in the QDA. The evaluated attributes were those selected by the panel of trained sensory panelists, among which the aromas of smoke and animal, which characterize off flavors, obtained averages below 1, which according to the CoEx [35] are considered absent and no difference was observed (*p* < 0.05) among the studied regions.

Cocoa, sweet and fruity attributes are clearly characterized in the samples while acidity and floral are present at a low intensity, while spices and woody traces were found, which may not be perceived if tasted again [35]. For the main characterized aromas, sweet did not show difference (*p* < 0.05) between the regions; for cocoa, the West region obtained the highest average differing from the regions of Lower Tocantins River and Trans-Amazon; while for fruity, the regions Lower Tocantins River and Northeast differed from each other, having the lowest and highest means, respectively, but they did not differ from the other regions.

The cocoa attribute is related to the typical flavor of roasted cocoa beans that are well fermented, dry and free of defects, and the sweet attribute is typically perceived in foods such as candies and desserts that contain sugar and also naturally found in fruits. Fruitiness is a characteristic of fresh fruits and brown fruits [36,37].

In a study with Brazilian chocolates, Cemin et al. [12] obtained the cocoa attribute as one of the highlights in the sensory evaluation, being clearly identified in the samples; in addition, the attributes acidity, woody and floral were identified in lower intensities, as well as in this research.

For flavor, sensory panelists identified 10 attributes that are shown in Table 3, such as mean intensity values obtained in the QDA by region. The attributes bitterness, cocoa and astringency are clearly identified in the samples, while acidity, sweetness and fruitiness are present in lower intensities. The floral, wood and spices traces were perceived, and smoke less than 1 is considered absent [35]. The Southeast region had the highest average for the floral attribute, differing significantly from the Trans-Amazon region with the lowest average. The Lower Tocantins River, Northeast and Southeast regions presented beans with more spice and wood flavor than the West and Trans-Amazon regions, differing significantly.

For the bitter attribute, the results obtained by the panel are consistent with the chemical data, since the regions of Lower Tocantins River and Southeast have higher and lower levels of total phenolic compounds (Table 4), respectively. These compounds are directly linked to the bitterness attribute according to Andrade et al. [38]; consequently, the beans from Lower Tocantins River are more bitter than those from the Southeast.

Sukha, Umaharan and Butler [39] suggested in a study carried out with cocoa from Trinidad that this plantation may follow the concept of “terroir”, where environmental factors and processing practices contribute to the unique characteristics of products of cocoa. The small variations in the attributes of cocoa beans from the 2019 harvest in Pará regions may be the result of the combination of all factors (exogenous and endogenous) that vary during a given year and type of culture that may affect final flavor development within different cocoa genotypes, as previously reported by Sukha et al. [40].

In this research, only five of the nine aroma attributes showed a significant difference between the regions (Table 2) and for the ten flavor attributes raised by the sensory team, only three showed a difference between the regions (Table 3); however, there was no highlight of any region, and moreover, they were in the same range of perception [35]. Indeed, other sensory profile studies also did not vary significantly across geographic origins for products such as apples [41], coffee [42] and cocoa from Colombia [43].

**Figure 1:**
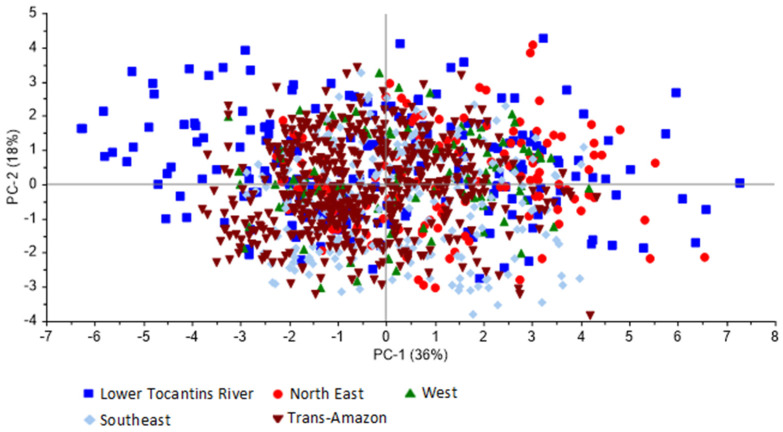
Principal component analysis of Quantitative Descriptive Analysis for aromas of Amazonian cocoa beans, presented by the score graph.

**Figure 2:**
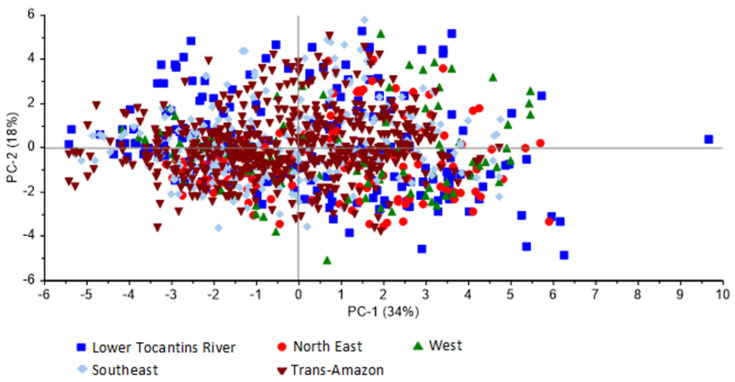
Principal component analysis of Quantitative Descriptive Analysis for Amazon cocoa bean flavors, presented by the score chart.

To verify the possibility of grouping bean samples by region according to their similarities and differences (Figure 1 and Figure 2), principal component analysis was performed with the sensory profile data obtained through QDA.

Figure 1 and Figure 2 reveal the relative contribution of each variable in each principal component (PC). It is observed that in Figure 1, PC1 and PC2 explained 54% of the data variance, while in Figure 2, PC1 and PC2 explained 52% of the data variance. In both, no cluster was formed; therefore, it is not possible to distinguish the origins of the samples. In the loading graphs (not shown) there were no differences between the regions, evidenced later in Figure 1 and Figure 2. The descriptors were also evaluated individually using PCA, but there was no cluster formation for aromas or flavors.

The best quality cocoa beans of recognized origin are more expensive, and the ability to identify their real geographic origin is desirable for consumers, producers, industries and authorities. Cocoa beans from the five main producing regions in the Eastern Amazon have a very similar sensory profile. For the 2019 harvest that was evaluated, this can be seen as a positive point, because regardless of the region, the Eastern Amazon presents a single profile; however, changes between harvests can occur due to environmental factors as well as changes in processing.

### 3.3. Acceptance Test

**Figure 3:**
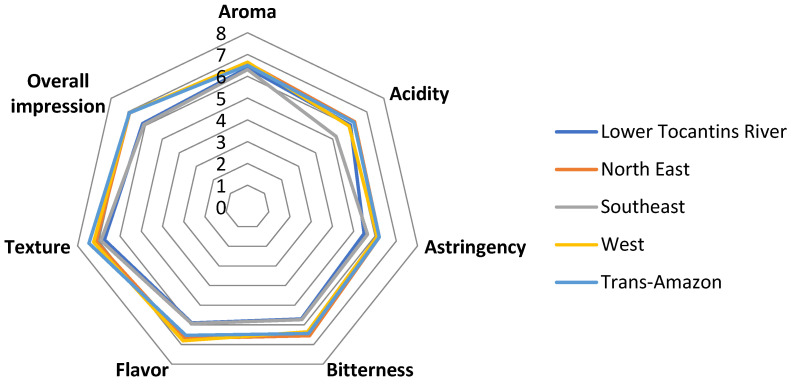
Evaluation of tasters’ preference obtained in the acceptance test of chocolates from the Amazonian regions.

The acceptance test was carried out to evaluate the chocolates made with beans from each region, as described in point 2.5. Figure 3 shows the tasters’ preference obtained in the acceptance test of the five chocolate samples.

The chocolates from the regions did not show very distant acceptance means, the Lower Tocantins River and Southeast regions had the lowest acceptance values in most of the evaluated attributes, and the points between these two regions overlap, demonstrating similar acceptance. The Northeast, West, and Trans-Amazon regions presented a higher acceptance index in most of the evaluated attributes; however, the Trans-Amazon region obtained a better acceptance.

The chocolate samples in general had greater acceptance only in the texture attribute with an average of 7 for some chocolates. Perez et al. [44] claim that dark chocolate is still significantly less popular than milk, despite all the appeal regarding the various health benefits of chocolate with high percentages of cocoa.

### 3.4. Sensory Profile of Cocoa Beans from the Main Producing Regions in the Eastern Amazon 

**Figure 4:**
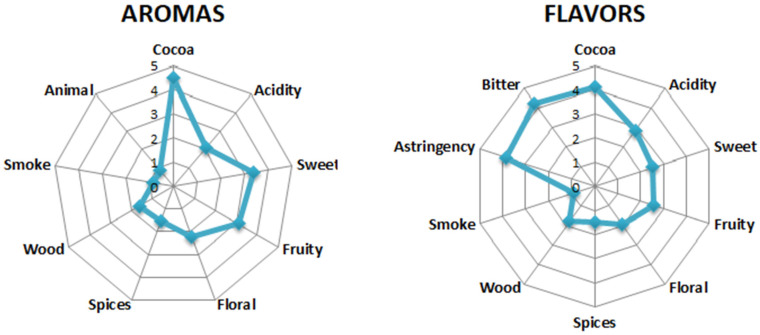
Sensory profile of aroma and flavor of Amazonian cocoa beans.

Figure 4 presents the sensory profiles of the aroma and flavor of Amazon cocoa beans, corresponding to the average of the results of the five evaluated regions. The zero point on the scale is the center, and intensity increases toward the ends of the axes. The average of each attribute for each product is plotted on the corresponding axis.

Among the aromas of Amazonian cocoa beans, it can be seen (Figure 4) that cocoa, sweet and fruity obtained the highest averages, while for flavor attributes, bitterness and astringency are more present in beans, as they are related to the levels of polyphenols and generally represent binding with weak fermentation [13]. The intensity of bitterness and astringency attributes can mask other characteristics such as floral and fruity.

### 3.5. Bioactive Compounds and Astringence 

In Table 4, the results of polyphenols ranged between 21.48 and 14.73 (mgCE/g), from the Lower Tocantins River and Southeast regions, respectively, despite the regions showing no difference (*p* < 0.05). Lower Tocantins River has higher levels of polyphenols, and this may be linked to geographic factors; due to the ecological processes of flooding that drive the exchange of water, materials and organisms between the flooded soil and the main course of the river; trophic interactions; and biodiversity, generating a soil richer in nutrients, directly benefiting the plantation [45,46].

Herman et al. [47] carried out a study with different drying times, temperatures and air speeds of beans from the Northeast region that were fermented for 7 days, where 16.05 (mgGAE/g) was obtained for polyphenols. In this research, the content found for the same region is 16.17 (mgCE/g), close to that found by the cited authors, despite the different fermentation, drying and standard analysis methods. 

The results of proanthocyanidins demonstrate that the Lower Tocantins River region has a high content of the compound, with a significant difference in relation to the Southeast, West and Trans-Amazon regions. Dang and Nguyen [48] carried out a study on the effects of maturity in harvesting and fermentation conditions on the bioactive compounds of cocoa beans with adequate maturation and obtained results of 9.04 (mgCE/100 g) for fermented beans and up to 27.28 (mgCE/100 g) for unfermented ones, demonstrating that the proanthocyanidin content varies according to fermentation time.

Polyphenolic compounds, such as proanthocyanidins (tannins), form complexes with salivary proteins and mucopolysaccharides. Protein–tannin complexes result in the precipitation and/or aggregation of salivary proteins, causing them to lose their lubricating properties and the mouth to experience a dry sensation called astringency [24]. 

The results of this research did not obtain a correlation between astringency and proanthocyanidins since the Southeast region that presented the lowest value of the compound had the second highest value for astringency of 47.84, a value inferior only to Lower Tocantins River, and both regions showed a difference (*p* < 0.05) from the others. 

## 4. Conclusions

Samples of commercial Amazon cocoa beans produced in the five evaluated regions show a satisfactory commercial standard in accordance with the legislation. 

Nine aroma and ten flavor attributes were selected in Amazonian cocoa beans and the Quantitative Descriptive Analysis found significant differences in some of these attributes between the regions. However, beans from the five main producing regions presented a very similar sensory profile for the 2019 harvest.

Chocolates produced with beans from the Trans-Amazon and Northeast regions were more preferred, with a strong influence of acidity, bitterness, astringency and flavor on global acceptance.

The cocoa beans used in this study showed differences in terms of chemical composition, in relation to proanthocyanidins and astringency, and the levels of phenolic compounds were consistent with those reported in the literature.

This work allowed for the establishment of the sensory and quality profile of commercial cocoa beans from the Eastern Amazon.

## Figures and Tables

**Table 1:** Mean results related to quality parameters evaluated in the bean cutting test and cocoa bean nib moisture by region.

Parameters	Region				
	Lower Tocantins River	Northeast	Southeast	West	Trans-Amazon
Browns (%)	25.17 ± 20.54 ^a^	24.75 ± 17.81 ^a^	61.33 ± 20.92 ^b^	57.27 ± 21.33 ^b^	49.22 ± 16.47 ^ab^
Partially Brown (%)	71.28 ± 20.52 ^a^	70.7 ± 19.20 ^a^	36.00 ± 17.70 ^a^	41.60 ± 22.17 ^a^	49.33 ± 16.70 ^a^
Violet (%)	1.22 ± 0.96 ^a^	1.00 ± 1.12 ^a^	1.22 ± 2.12 ^a^	0.24 ± 0.63 ^a^	0.20 ± 0.48 ^a^
White (%)	0.28 ± 0.53 ^a^	0.92 ± 0.92 ^a^	0.78 ± 1.07 ^a^	0.44 ± 0.31 ^a^	0.80 ± 1.13 ^a^
CC (%)	86.50 ± 16.30 ^a^	91.42 ± 5.66 ^a^	96.89 ± 2.83 ^a^	90.7 ± 10.85 ^a^	93.51 ± 8.13 ^a^
SC (%)	13.39 ± 16.39 ^a^	8.42 ± 5.83 ^a^	2.78 ± 2.83 ^a^	9.28 ± 10.85 ^a^	6.20 ± 8.22 ^a^
Defects (%)	1.76 ± 0.44 ^a^	0.97 ± 0.32 ^ab^	0.6 ± 0.12 ^ab^	0.18 ± 0.05 ^b^	0.19 ± 0.04 ^b^
Moisture (%)	6.11 ± 0.63 ^a^	5.42 ± 0.37 ^a^	5.47 ± 0.48 ^a^	5.76 ± 0.31 ^a^	5.93 ± 0.54 ^a^
Acidity (g Aa/100 g)	0.21 ± 0.08 ^a^	0.33 ± 0.09 ^ab^	0.44 ± 0.13 ^ab^	0.48 ± 0.27 ^b^	0.47 ± 0.14 ^b^
Aw	0.62 ± 0.06 ^a^	0.58 ± 0.08 ^a^	0.59 ± 0.02 ^a^	0.61 ± 0.03 ^a^	0.62 ± 0.04 ^a^

CC—beans with compartmentation; SC—beans without division; g Aa/100 g—gram of acetic acid per 100 g of dry nibs; Aw—water activity. Results expressed as mean ± standard deviation. Means with letters in common, in the same row, do not show a significant difference (*p* < 0.05) by Tukey’s test.

**Table 2:** Average values of intensity of sensory attributes of aromas of Amazonian cocoa beans by region.

	Region				
Aromas	Lower Tocantins River	Northeast	Southeast	West	Trans-Amazon
Cocoa	4.13 ± 0.64 ^a^	4.69 ± 0.41 ^ab^	4.49 ± 0.27 ^ab^	4.85 ± 0.32 ^b^	4.27 ± 0.22 ^a^
Acidity	2.05 ± 0.23 ^ab^	2.20 ± 0.20 ^ab^	2.27 ± 0.13 ^a^	2.18 ± 0.22 ^a^	1.84 ± 0.24 ^b^
Sweet	3.08 ± 0.63 ^a^	3.83 ± 0.17 ^a^	3.43 ± 0.30 ^a^	3.39 ± 0.40 ^a^	3.17 ± 0.41 ^a^
Fruity	2.69 ± 0.74 ^a^	3.73 ± 0.63 ^b^	2.99 ± 0.30 ^ab^	3.19 ± 0.18 ^ab^	2.93 ± 0.51 ^ab^
Floral	2.21 ± 0.47 ^a^	2.60 ± 0.33 ^a^	2.17 ± 0.22 ^a^	2.17 ± 0.21 ^a^	1.99 ± 0.45 ^a^
Spices	1.80 ± 0.20 ^a^	1.86 ± 0.18 ^a^	1.71 ± 0.14 ^a^	1.18 ± 0.24 ^b^	1.11 ± 0.15 ^b^
Wood	1.83 ± 0.29 ^a^	2.00 ± 0.38 ^a^	1.80 ± 0.84 ^ab^	1.27 ± 0.20 ^b^	1.30 ± 0.13 ^b^
Smoke	0.76 ± 0.28 ^a^	0.96 ± 0.05 ^a^	0.79 ± 0.19 ^a^	0.83 ± 0.39 ^a^	0.82 ± 0.20 ^a^
Animal	0.77 ± 0.30 ^a^	1.06 ± 0.11 ^a^	0.76 ± 0.27 ^a^	0.85 ± 0.24 ^a^	0.86 ± 0.15 ^a^

Mean ± standard deviation results, obtained using a 9 cm unstructured scale. Means with letters in common, in the same row, do not show a significant difference (*p* < 0.05) by Tukey’s test.

**Table 3:** Mean intensity values of Amazonian cocoa flavor sensory attributes by region.

Flavors	Region				
	Lower Tocantins River	Northeast	Southeast	West	Trans-Amazon
Cocoa	3.88 ± 0.67 ^a^	4.39 ± 0.27 ^a^	4.36 ± 0.13 ^a^	4.10 ± 0.42 ^a^	3.88 ± 0.28 ^a^
Acidity	2.25 ± 0.43 ^a^	3.18 ± 0.45 ^a^	3.49 ± 1.24 ^a^	2.81 ± 0.84 ^a^	2.58 ± 0.59 ^a^
Sweet	2.42 ± 0.67 ^a^	2.94 ± 0.23 ^a^	2.58 ± 0.23 ^a^	2.16 ± 0.34 ^a^	2.50 ± 0.44 ^a^
Fruity	2.40 ± 0.39 ^a^	3.07 ± 0.53 ^a^	2.88 ± 0.38 ^a^	2.23 ± 0.36 ^a^	2.42 ± 0.53 ^a^
Floral	1.92 ± 0.53 ^ab^	2.14 ± 0.31 ^ab^	2.35 ± 0.08 ^b^	1.79 ± 0.36 ^ab^	1.66 ± 0.28 ^a^
Spices	1.72 ± 0.16 ^a^	1.75 ± 0.09 ^a^	1.71 ± 0.26 ^a^	1.01 ± 0.17 ^b^	1.16 ± 0.19 ^b^
Wood	2.00 ± 0.23 ^a^	2.21 ± 0.23 ^a^	1.84 ± 0.37 ^a^	1.35 ± 0.22 ^a^	1.38 ± 0,17 ^b^
Smoke	0.88 ± 0.29 ^a^	1.03 ± 0.09 ^a^	0.94 ± 0.46 ^a^	0.87 ± 0.31 ^a^	0.92 ± 0.19 ^a^
Astringency	3.79 ± 0.59 ^a^	3.85 ± 0.28 ^a^	4.09 ± 0.58 ^a^	3.68 ± 0.36 ^a^	3.86 ± 0.49 ^a^
Bitter	4.73 ± 0.41 ^a^	4.06 ± 0.12 ^a^	3.97 ± 0.40 ^a^	4.11 ± 0.46 ^a^	4.23 ± 0.50 ^a^

Results expressed as mean ± standard deviation, obtained using a 9 cm unstructured scale. Means with letters in common, in the same row, do not show a significant difference (*p* < 0.05) by Tukey’s test.

**Table 4:** Mean results of bioactive compounds and astringency of bean nibs by region.

Region	Total Polyphenols (mgCE/g)	Proanthocyanidins (mgCyE/g)	Astringency (NTU/g)
Lower Tocantins River	21.48 ± 13.28 ^a^	23.53 ± 9.13 ^a^	48.68 ± 6.93 ^a^
Northeast	16.17 ± 3.24 ^a^	15.80 ± 8.04 ^ab^	34.31 ± 1.26 ^b^
Southeast	14.73 ± 5.96 ^a^	7.32 ± 4.20 ^b^	47.84 ± 10.26 ^a^
West	14.90 ± 5.80 ^a^	11.24 ± 5.50 ^b^	31.32 ± 5.06 ^b^
Trans-Amazon	18.88 ± 6.56 ^a^	11.97 ± 7.52 ^b^	27.26 ± 3.32 ^b^

Results expressed as mean ± standard deviation. Means with letters in common, in the same column, do not show a significant difference (*p* < 0.05) by Tukey’s test. Polyphenols: mgCE/g—milligrams of catechin equivalent per gram. Proanthocyanidins: mgCyE/g—milligrams of cyanidin equivalent per gram. Astringency: NTUs—Nephelometric Turbidity Units.

## Data Availability

The original contributions presented in the study are included in the article, further inquiries can be directed to the corresponding author.
